# Supplementary material for: Eating disorders in weight-related therapy (EDIT): Protocol for a systematic review with individual participant data meta-analysis of eating disorder risk in behavioural weight management
Source: PLoS One. 2023 Jul 10;18(7):e0282401. doi: 10.1371/journal.pone.0282401 (PMC10332604; doi:10.1371/journal.pone.0282401)
Supplement: S1 Table — (PDF) [file pone.0282401.s001.pdf]

Table S1: Search strategies

MEDLINE

1. exp Obesity/
2. exp Overweight/
3. obes*.tw.
4. overweight.tw.
5. 1 or 2 or 3 or 4
6. weight loss/
7. exp diet therapy/
8. exp bariatrics/
9. exp exercise/
10. anti-obesity agents/ or appetite depressants/
11. (diet* adj2 therap*).tw.
12. bariatric*.tw.
13. (low adj3 (energy or calor*) adj4 diet).tw.
14. ((pharma* or diet* or obes* or lifestyle or behavio*) adj3 (interven* or treat* or therap*)).tw.
15. ((calori* or diet*) adj3 (reduc* or restrict*)).tw.
16. (weight adj3 (manag* or los*)).tw.
17. (exercis* or physical activit*).tw.
18. HAES.mp.
19. health at every size.mp.
20. (weight adj2 neutral).mp.
21. nondiet.mp.
22. (non adj2 diet).mp.
23. (intuitive adj2 eat*).mp.
24. mindful*.tw.
25. 6 or 7 or 8 or 9 or 10 or 11 or 12 or 13 or 14 or 15 or 16 or 17 or 18 or 19 or 20 or 21 or 22 or 23 or 24
26. Body Image/
27. (body adj3 (accept* or dissatisf* or image or satisf* or appreciat* or esteem)).tw.
28. "feeding and eating disorders"/ or anorexia nervosa/ or binge-eating disorder/ or bulimia nervosa/ or "feeding and eating disorders of childhood"/
29. (bulimi* adj3 symptom*).tw.
30. (disorder* adj3 eat*).tw.
31. (emotion* adj3 eat*).tw.
32. (diet* adj3 restr*).tw.
33. (binge adj3 eat*).tw.
34. extreme weight loss.tw.
35. loss of control.tw.
36. drive for thinness.tw.
37. ((weight or shape or eat*) adj3 concern).tw.
38. (eat* adj2 behavi*).tw.
39. 26 or 27 or 28 or 29 or 30 or 31 or 32 or 33 or 34 or 35 or 36 or 37 or 38
40. randomized controlled trial/
41. (randomi?ed controlled trial* or RCT* or (controlled adj3 trial)).mp.
42. randomi?ed.ti.
43. clinical trials as topic.sh.
44. randomly.ab.
45. trial.mp.
46. clinical trial.mp.
47. 40 or 41 or 42 or 43 or 44 or 45 or 46
48. 5 and 25 and 39 and 47

EMBASE

1. obesity/
2. obes*.tw.
3. overweight.tw.
4. 1 or 2 or 3
5. weight reduction/

6. diet therapy/ or diet restriction/ or low calory diet/ or low fat diet/
7. bariatric surgery/ or gastric banding/ or sleeve gastrectomy/
8. exercise/
9. antiobesity agent/
10. (diet* adj2 therap*).tw.
11. bariatric*.tw.
12. (low adj4 (energy or calor*) adj4 diet).tw.
13. ((pharma* or diet* or obes* or lifestyle or behavio*) adj3 (interven* or treat* or therap*)).tw.
14. ((calori* or diet*) adj3 (reduc* or restrict*)).tw.
15. (weight adj3 (manag* or los*)).tw.
16. (exercis* or physical activit*).tw.
17. HAES.mp.
18. health at every size.mp.
19. (weight adj2 neutral).mp.
20. nondiet.mp.
21. (non adj2 diet).mp.
22. (intuitive adj2 eat*).mp.
23. mindful*.tw.
24. 5 or 6 or 7 or 8 or 9 or 10 or 11 or 12 or 13 or 14 or 15 or 16 or 17 or 18 or 19 or 20 or 21 or 22 or 23
25. body image/
26. (body adj3 (accept* or dissatisf* or image or satisf* or appreciat* or esteem)).tw.
27. eating disorder/ or anorexia nervosa/ or binge eating disorder/ or bulimia/
28. feeding behavior/
29. (bulimi* adj3 symptom*).tw.
30. (disorder* adj3 eat*).tw.
31. (emotion* adj3 eat*).tw.
32. (diet* adj4 restrain*).tw.
33. (binge adj3 eat*).tw.
34. extreme weight loss.tw.
35. loss of control.tw.
36. drive for thinness.tw.
37. ((weight or shape or eat*) adj3 concern).tw.
38. 25 or 26 or 27 or 28 or 29 or 30 or 31 or 32 or 33 or 34 or 35 or 36 or 37
39. randomized controlled trial/ or controlled clinical trial/
40. (randomi?ed controlled trial* or RCT* or (controlled adj3 trial)).mp.
41. randomi?ed.ti.
42. randomly.ab.
43. trial.mp.
44. clinical trial.mp.
45. 39 or 40 or 41 or 42 or 43 or 44
46. 4 and 24 and 38 and 45

PsycINFO

1. Obesity/
2. Overweight/
3. obes*.tw.
4. overweight.tw.
5. 1 or 2 or 3 or 4
6. weight loss/ or weight control/
7. diets/
8. exp bariatric surgery/
9. exp exercise/
10. (diet* adj2 therap*).tw.
11. bariatric*.tw.
12. (low adj3 (energy or calor*) adj4 diet).tw.
13. ((pharma* or diet* or obes* or lifestyle or behavio*) adj3 (interven* or treat* or therap*)).tw.
14. ((calori* or diet*) adj3 (reduc* or restrict*)).tw.
15. (weight adj3 (manag* or los*)).tw.

16. exercis*.mp. or physical activit*.tw.<br>17. HAES.mp.<br>18. health at every size.mp.<br>19. (weight adj2 neutral).mp.<br>20. nondiet.mp.<br>21. (non adj2 diet).mp.<br>22. (intuitive adj2 eat*).mp.<br>23. mindful*.tw.<br>24. 6 or 7 or 8 or 9 or 10 or 11 or 12 or 13 or 14 or 15 or 16 or 17 or 18 or 19 or 20 or 21 or 22 or 23<br>25. Body Image/<br>26. (body adj3 (accept* or dissatisf* or image or satisf* or appreciat* or esteem)).tw.<br>27. eating disorders/ or anorexia nervosa/ or binge eating disorder/ or bulimia/ or hyperphagia/ or "purging (eating disorders)"/<br>28. eating behavior/ or binge eating/ or dietary restraint/<br>29. (bulimi* adj3 symptom*).tw.<br>30. (disorder* adj3 eat*).tw.<br>31. (emotion* adj3 eat*).tw.<br>32. (diet* adj3 restr*).tw.<br>33. (binge adj3 eat*).tw.<br>34. extreme weight loss.tw.<br>35. loss of control.tw.<br>36. drive for thinness.tw.<br>37. ((weight or shape or eat*) adj3 concern).tw.<br>38. 25 or 26 or 27 or 28 or 29 or 30 or 31 or 32 or 33 or 34 or 35 or 36 or 37<br>39. randomized controlled trials/ or clinical trials/ or randomized clinical trials/<br>40. (randomi?ed controlled trial* or RCT* or (controlled adj3 trial)).mp.<br>41. randomi?ed.ti.<br>42. randomly.ab.<br>43. trial.mp.<br>44. clinical trial.mp.<br>45. 39 or 40 or 41 or 42 or 43 or 44<br>46. 5 and 24 and 38 and 45
**SCOPUS**<br>(TITLE-ABS-KEY ("clinical trials" OR "clinical trials as a topic" OR "randomized controlled trial" OR "Randomized Controlled Trials as Topic" OR "controlled clinical trial" OR "Controlled Clinical Trials as Topic" OR "Clinical trial*" OR trial* OR rct OR random*)) AND ((TITLE-ABS-KEY (obes* OR overweight*)) AND ((TITLE-ABS-KEY ("Weight loss" OR diet* OR bariatric* OR exercis* OR "anti-obesity agent*" OR haes OR "health at every size" OR "Weight neutral" OR "Intuitive eat*" OR mindful*)) OR (TITLE-ABS-KEY (((pharma* OR diet* OR obes* OR lifestyle OR behavio*) W/4 (interven* OR treat* OR therap*)))))) AND ((TITLE-ABS-KEY (((weight OR shape OR eat*) W/3 concern*))) OR ((TITLE-ABS-KEY ("Body image*" OR "Eating disorder*" OR anorexia OR "binge eating disorder*" OR bulimi* OR "Emotion* eat*" OR "Diet* restr*" OR "Binge eat*" OR "extreme weight loss*" OR "loss of Control" OR "Drive for thinness")) OR (TITLE-ABS-KEY (((weight OR shape OR eat*) W/3 concern*))))))
**Clinicaltrials.gov**<br>Key search terms via basic search platform:<br>(weight management OR obesity treatment)
**WHO ICTRP**<br>Key search terms via basic search platform:<br>(weight management OR obesity treatment)
