# Supplementary material for: Eating disorders in weight-related therapy (EDIT): Protocol for a systematic review with individual participant data meta-analysis of eating disorder risk in behavioural weight management
Source: PLoS One. 2023 Jul 10;18(7):e0282401. doi: 10.1371/journal.pone.0282401 (PMC10332604; doi:10.1371/journal.pone.0282401)
Supplement: S2 Table — (PDF) [file pone.0282401.s002.pdf]

Table S2: Randomised controlled trails eligible for inclusion in the Eating Disorders In weight-related Therapy (EDIT) Collaboration

This table includes all trials that we have identified up to August 2022 as being eligible for inclusion in EDIT. Trials shaded in blue have agreed to join the Collaboration and share data. Trials shaded in yellow no longer have individual participant data available to share.

Country/ citation	Trial registration number	Primary contact	Start/ end recruitment period (years)	Sample size	Population	Eating disorder tool/s
**ADOLESCENT TRIALS**						
Australia, Bonham et al. 2017(1)	ISRCTN13602313	Maxine Bonham & Aimee L. Dordevic	2013/2015	74	13-17y, BMI z-score ≥1.282, >85th percentile	ChEDE, EDE-Q
Australia, Brennan et al. 2012(2)	ACTRN12610000111077			63	11-19y, overweight or obesity	EDI-2
Australia, Lister et al. 2020(3)	ACTRN12617001630303	Natalie B Lister	2018/2023	186	13-17y, adult equivalent BMI > 30 kg/m^2	EDE-Q, BES
Australia, Partridge et al. 2020(4)	ACTRN12619000389101	Stephanie Partridge	2020/2022	150	13 -18y, adult equivalent BMI 25.0-29.9 kg/m^2	EDE-Q
Australia, Williams et al.	ACTRN12611000139976		2011-2014	570	12-17 years; >= 85th centile for age and gender specific BMI data.	EDE-Q
Belgium, Braet et al. 2000(5)		Caroline Braet	1989/1991	136	7-17y, seeking treatment for obesity	EDI
Belgium, Braet et al. 2004(6)		Caroline Braet	1996/1999	122	7-17y, BMI > 95th percentile	EDE, EDI
Belgium, Desmet et al.	ISRCTN47384427		2021/ongoing	70	12-16 years; >140% overweight.	ChEDE-Q
Belgium, Naets et al. 2018(7)	ISRCTN14722584	Annelies Van Eyck	2017/2019	200	8-18y, overweight or obesity	EDE
Brazil, Lofrano-Prado & Lo Prado et al.(8)		Wagner Prado & Mara Lofrano-Prado		62	13-18y BMI >95th percentile	EAT, BITE
Brazil, Lofrano-Prado et al. 2021(9)		Wagner Prado & Mara Lofrano-Prado		74	13-18y, BMI z-score ≥ 2.0	BES, EAT-26, BITE
Brazil, Samara Audi et al.	RBR-7bxzh2r		2018/ongoing	60	13-17 years; BMI z-score greater than or equal to 2	BES
Germany, Blüher et al. 2014(10)	DRKS00005299		2013/2014	65	8-18 years; BMI > 97th percentile.	ChEDE-Q
Netherlands, Jansen et al. 2011(11)		Elena Jansen		98	≥130% overweight	EDE-Q
Norway, Skjåkødegård et al. 2016(12)	NCT02687516	Hanna F Skjakodegard & Yngvild S Danielsen	2014/2018	120	6-18y, BMI ≥ 35, or ≥ 30 with obesity related co-morbidity	YEDE-Q

Portugal, Ramalho et al. 2020(13)	NCT04642222	Sofia Ramalho		210	13-18y, BMI ≥ 85th percentile	ChEAT
UK, Croker et al. 2012(14)	ISRCTN 51382628.	Dasha Nicholls	2004/2008	72	8-12y, overweight or obesity	ChEAT
USA, Boutelle et al. 2011(15)		Kerri Boutelle		36	8-12y, BMI ≥ 85th percentile and child eating in the absence of hunger	ChEDE, BES
USA, Cardel, Newsome et al. 2022	NCT04484831	Michelle Cardel & Faith Newsome	2020/2021	40	14-19y, BMI ≥85th percentile for sex and age	EAT-26
USA, Darling et al. 2021(16)	NCT02426436			66	13-17y, BMI >85th percentile and an absolute BMI <50 kg/m^2	EDE-Q
USA, DeBar et al. 2012(17)	NCT01068236	Lynn DeBar	2005/2009	208	12-17y, BMI ≥ 90th percentile	QEWP-Adolescent
USA, Douglas et al. 2020(18)	NCT04027426	Hollie Raynor	2019	156	8-12 years, ≥ 85th percentile BMI, one adult caregiver with a BMI ≥ 25 kg/m2.	KEDS
USA, Doyle et al. 2008(19)		Andrea Goldschmidt	2003/2005	80	12-18y, overweight, ≥85th percentile	EDE-Q
USA, Eichen et al. 2019(20)	NCT01197443	Dawn Eichen & Kerri Boutelle		150	8-12y, BMI percentile between 85 and 99.9), one parent with BMI >25 kg/m^2	EDE-Q
USA, Epstein et al. 2001(21)		Denise E Wilfley		47	8-12y, 20% - 100% overweight compared to 50th percentile	BES, KEDS
USA, Estabrooks et al. 2009(22)	NCT00433901		2004/2006	220	8–12y, BMI ≥ 85th percentile	KEDS
USA, Follansbee-Junger et al. 2010(23)			2006/2008	68	8-13y, overweight or obesity	ChEAT
USA, Goldschmidt et al. 2014(24)		Denise E Wilfley		150	7-12y, 20–100% above median BMI, with at least one parent with BMI≥25 kg/m^2	ChEDE, EDE-Q
USA, Jelalian et al. 2006(25)			2000/2002	76	13-16y, 20- 80% overweight	BES
USA, Raynor et al. 2021*(26)	NCT02586090	Melanie Bean	2016/2018	82	12-16y, BMI ≥ 85th percentile	EDE-Q
USA, Rhee et al.	NCT02976636	Kay (Kyung) Rhee	2017/2022	160	7 - 12 y, BMI ≥ 85th but <100% overweight; at least one parent with (BMI≥25)	
USA, Saelens et al. 2002(27)				44	12-16y, 20% - 100% above the median (50th percentile)	ChEAT

USA; Sato et al.	NCT04038684			240	13-18 years, ≥ 85th%ile for age and sex	YEDE-Q
USA, Shomaker et al. 2017(28)	NCT00263536	Marian Tanofsky-Kraff & Jack Yanovski	2012/2014	29	8-13y, BMI ≥ 85th percentile, one parent with overweight/ obesity (BMI ≥ 25 kg/m^2)	ChEDE, QEWP-Adolescent
USA, Vidmar et al. 2020(29)	NCT03954223	Alaina Vidmar		60	14-18y, BMI ≥ 95th percentile	BEDS-7
**ADULT TRIALS**						
Australia, Cheng et al. 2014(30)	ACTRN12609000307202	Hoi Lun Cheng	2006/2009	71	18–25y, BMI ≥ 27.5 kg/m^2	BES
Australia, Raman et al. 2018(31)	ACTRN12613000537752	Evelyn Smith	2013/2014	80	18-55y, BMI > 30 kg/m^2, weight < 180 kg	EDE-Q
Australia, Rieger et al. 2017(32)	ACTRN12611000509965	Elizabeth Rieger	2010/2013	201	18-65y, BMI ≥ 30 kg/m^2	BES, EAT
Australia, Seimon et al. 2019(33)	12612000651886	Amanda Salis	2013/2016	101	45-65y, postmenopausal women, BMI 30-40 kg/m^2, sedentary	EDE, EDE-Q
Australia, Smith et al. 2017(34)	ACTRN12616000658415	Evelyn Smith		176	18–55y, BMI ≥ 30.0 kg/m^2	EDE-Q
Australia, Zwickert et al. 2016(35)		Elizabeth Rieger	2013/2015	60	18-65y, BMI ≥ 30.0 kg/m^2	BES
Brazil, Bolognese et al. 2020(36)	RBR-2YZS7	Braulio Henrique Magnani Branco		74	40–59y, BMI ≥ 25 kg/m^2, female	EAT-26
Brazil, Salvo et al. 2018(37)	NCT02893150	Vera Salvo & Marcelo Demarzo		240	18-60y, BMI 25-40 kg/m^2, female	BES, EAT
Canada, Moss et al. 2017(38)	NCT02649634		2007/2010	135	BMI >= 25 kilograms, 18 Years+	EDE-Q
Canada, Tanco et al. 1998(39)		Wolfgang Linden		62	≥19y, BMI ≥ 30 kg/m^2, female	EDI
Finland, Fogelholm et al. 1999(40)		Mikael Fogelholm		85	29-46y, premenopausal, BMI 29-46 kg/m^2, female	BITE
Finland, Keränen et al. 2009(41)			2002/2004	82	18–65y, BMI > 27 kg/m^2	BES
Germany, Hilbert et al. 2016(42)	DRKS00005182	Anja Hilbert	2013/2017	72	≥18y, BMI 25-45kg/m^2	EDE-Q8
Greece, Christaki et al. 2013(43)	UoAMedPR-4716-180211-25		2010/2011	34	BMI > 28 kg/m^2, female	EAT-26
Italy, Dalle Grave et al. 2013(44)	USL22#01/07-CEP31	Riccardo Dalle Grave & Simona Calugi		88	18-65y, BMI ≥ 40.0 kg/m^2 or 35-39.9 kg/m^2 with at least one comorbidity	BES

Italy, Muggia et al. 2014(45)	NCT01686854		2007/2011	163	18-65y, BMI 25-39.9 kg/m^2	BITE 16
New Zealand, Jospe et al. 2017(46)	ACTRN12615000010594	Rachael W. Taylor	2014/2015	250	≥18y, BMI > 27 kg/m^2	EDE-Q
Netherlands, Boh et al. 2016(47)	NTR5473		2015/na	134	18-60y, BMI > 25 kg/m^2	EDE-Q
Netherlands, Dassen et al. 2018(48)		Katrijn Houben		91	18-60y, BMI > 25 kg/m^2	EDE-Q
Netherlands, Schyns et al. 2020(49)				45	18-60y, BMI > 27 kg/m^2, female	EDE-Q
Netherlands, Werrij et al. 2009(50)				204	18-65y, BMI > 27 kg/m^2	EDE-Q
Romania; Podina et al.(51)	ISRCTN70907354			74	18-35 years, BMI 25-29.9 kg/m2	Eating Disorders Beliefs Questionnaire
Spain, Félix et al.	NCT03937167		2017/2019	180	18-65 years. BMI >= 30	EDI
UK, Beaulieu et al. 2020(52)	NCT03447600	Kristine Beaulieu	2018/2018	46	18-55y, BMI 25.0-34.9 kg/m^2	BES
UK, Cooper et al. 2010(53)		Zafra Cooper		150	20-60y, BMI 30.0 - 39.9 kg/m^2, female	EDE
UK, Scott et al. 2019(54)	ISRCTN88405328		2017/2019	1627	≥18y, BMI > 25 kg/m^2, <150kg	BES
UK, Simpson et al. 2015(55)	ISRCTN35774128	Sharon Simpson	2011/2012	170	18–70y, BMI of ≥ 30 kg/m^2	EDE-Q
UK, Whitelock et al. 2019(56)	NCT03602001	Victoria Whitelock & Eric Robinson	2017/20118	107	18–65y, BMI ≥25 kg/m^2	BES
USA, Afari et al. 2019(57)	NCT01757847			88	18–75y, BMI ≥ 25 kg m^2	BES
USA, Ariel et al. 2016(58)				612	21-75 years, BMI ≥ 30 & ≤ 45	BES
USA, Bacon et al. 2002(59)				78	30-45y, BMI 30-45 kg/m^2, female	EDI-2
USA, Barnes et al. 2017(60)	NCT02578199	Rachel Barnes		59	BMI 25-55 kg/m^2	EDE, EDE-Q
USA, Boutelle et al. 2019(61)		Kerri Boutelle	2015/2017	271	18–65y, BMI 25-45 kg/m^2	EDE, EDE-Q, BES
USA, Carels et al. 2014(62)				59	BMI ≥ 27 kg/m^2	BES
USA, Carels et al. 2019(63)				53	≥18y, BMI ≥ 27 kg/m^2	BES
USA, Carpenter et al. 2019(64)		Kelly Carpenter	2014/2015	75	≥18y, BMI 25-35 kg/m^2	BES

USA, Dennis et al. 1999(65)				39	Obesity	BES
USA, Dennis et al. 2001(66)				59	50-65y, BMI 27-40 kg/m^2	BES
USA, DiMarco et al. 2009(67)				39	18-55y, BMI 27 – 40 kg/m^2	EDE-Q
USA, Eichen et al.	NCT03724396	Dawn Eichen	2020/2021	67	18-65y, BMI >25 and ≤45	EDE, EDE-Q
USA, Glynn et al. 2022(68)	NCT03057873		2017/2018	206	25-50 y, BMO 27-35 inclusive, sans childbearing potential	BES
USA, Goodrick et al. 1998(69)				219	25-50y, 14 - 41 kgs overweight	BES
USA, Hilderbrandt et al.	NCT04797169		2021/2024 (predicted)	600	18-60 years, BMI > 27 kg/m2 & interested in the Noom app.	EDE-Q
USA, Jeffery et al. 1995(70)		Robert Jeffrey		122	120-140% of ideal weight	BES
USA, Jeffery et al. 1998(69)		Robert Jeffrey		193	25-55y, 14 - 32kg overweight	Gormally Binge Eating Questionnaire
USA, Kalarchian et al. 2013(71)	NCT00623792	Melissa Kalarchian		240	≥18y, seeking bariatric surgery	EDE
USA, LaRose et al. 2014(72)	NCT01096719	Hollie Raynor		178	≥21y, BMI 27–45 kg/m^2	EDDS
USA, Lillis et al. 2015(73)	NCT01461421			160	18–70y, BMI 25–50 kg/m^2	EDE-Q
USA, Martin et al. 2019(74)	NCT01264406	Corby K Martin	2010/2015	198	BMI 25-45 kg/m^2, sedentary	MAEDS
USA, Mason et al. 2019(75)	NCT00470119	Anne McTiernan		439	50–75y, BMI ≥25.0 kg/m^2 (if Asian-American BMI ≥23.0 kg/m^2) Female only.	BES
USA, Mensinger et al. 2016(76)	NCT00769717 NCT00769717	Janell Mensinger	2008/2011	80	30-45y, BMI 30 - 45 kg/m^2	EDE-Q
USA, Napolitano et al. 2017(77)	NCT02342912		2015/2017	450	18–35y, BMI of 25–45 kg/m^2	EDDS
USA, Pacanowski et al. 2014(78)		Carly Pacanowski & Nancy Sherwood	2007/2008	419	19-70y, achieved ≥ 10% weight loss	EDDS binge eating items
USA, Radin et al. 2020(79)	https://aspredicted.org/nk4av.pdf; #8472			194	≥18y, BMI 30-45.9 kg/m^2	BES
USA, Ramirez et al. 2001(80)		Elena Ramirez		65	≥18y, BMI > 27.3 kg/m^2 for women, > 27.8 kg/m^2 for men	EDE-Q

USA, Raynor et al. 2006(81)		Hollie Raynor		30	BMI 25 – 40 kg/m^2	BES
USA, Smith et al. 2018(82)	NCT02753972	Brian M Shelley	2006/2006	36	50–70y, postmenopausal, BMI > 30 kg/m^2	BES
USA, Steinberg et al. 2014(83)			2011/2011	91	18–60y, BMI 25–40 kg/m^2	QEWP-R, Mizes Anorectic Cognitions Questionnaire
USA, Stice et al.	NCT03375853		2017/2023 (predicted)	180	18-38 years, BMI 25-35	EDE
USA, Vander et al. 2006(84)				80	18-65y, BMI ≥ 30 kg/m^2	NESQ
USA, Varady et al.	NCT04692532	Krista Varady	2021/2023	90	18-70y, obesity	MAEDS
USA, Wadden et al. 1994(85)				49	≥25kg overweight	BES
USA, Wadden et al. 2004(86)				123	BMI 30–43 kg/m^2, female	EDE
USA, Williamson et al. 2008(87)		Corby K Martin		48	25-50y, BMI 25-30 +kg/m^2	MAEDS

Abbreviations: BEDS-7, Binge Eating Disorder Screener-7; BES, Binge Eating Scale; BITE, Bulimic Investigatory Test of Edinburgh; EAT, Eating Attitudes Test; EDDS, Eating Disorder Diagnostic Scale; EDE, Eating Disorder Examination; EDE-Q, Eating Disorder Examination Questionnaire; EDI, Eating Disorder Inventory; MAEDS, Multidimensional Assessment of Eating-Disorder Symptom; NESQ, Night Eating Syndrome Questionnaire; QEWP, Questionnaire of Eating and Weight Patterns.

* Trial includes adolescents and adult data

References

1. Bonham MP, Dordevic AL, Ware RS, Brennan L, Truby H. Evaluation of a Commercially Delivered Weight Management Program for Adolescents. The Journal of Pediatrics. 2017;185:73-80.e3.
2. Brennan L, Wilks R, Walkley J, Fraser SF, Greenway K. Treatment Acceptability and Psychosocial Outcomes of a Randomised Controlled Trial of a Cognitive Behavioural Lifestyle Intervention for Overweight and Obese Adolescents. Behaviour Change. 2012;29(1):36-62.
3. Lister NB, Jebeile H, Truby H, Garnett SP, Varady KA, Cowell CT, et al. Fast track to health — Intermittent energy restriction in adolescents with obesity. A randomised controlled trial study protocol. Obesity Research & Clinical Practice. 2020;14(1):80-90.
4. Partridge SR, Raeside R, Singleton AC, Hyun K, Latham Z, Grunseit A, et al. Text Message Behavioral Intervention for Teens on Eating, Physical Activity and Social Wellbeing (TEXTBITES): Protocol for a Randomized Controlled Trial. JMIR Res Protoc. 2020;9(2):e16481.
5. Braet C, Van Winckel M. Long-term follow-up of a cognitive behavioral treatment program for obese children. Behavior Therapy. 2000;31(1):55-74.
6. Braet C, Tanghe A, Decaluwé V, Moens E, Rosseel Y. Inpatient treatment for children with obesity: weight loss, psychological well-being, and eating behavior. J Pediatr Psychol. 2004;29(7):519-29.
7. Naets T, Vervoort L, Ysebaert M, Van Eyck A, Verhulst S, Bruyndonckx L, et al. WELCOME: improving WEight controL and CO-Morbidities in children with obesity via Executive function training: study protocol for a randomized controlled trial. BMC Public Health. 2018;18(1):1075.
8. de Lira CT, Dos Santos MA, Gomes PP, Fidelix YL, Dos Santos AC, Tenório TR, et al. Aerobic training performed at ventilatory threshold improves liver enzymes and lipid profile related to non-alcoholic fatty liver disease in adolescents with obesity. Nutr Health. 2017;23(4):281-8.
9. Lofrano-Prado MC, Donato Junior J, Lambertucci AC, Lambertucci RH, Malik N, Ritti-Dias RM, et al. Recreational Physical Activity Improves Adherence and Dropout in a Non-intensive Behavioral Intervention for Adolescents with Obesity. Res Q Exerc Sport. 2021:1-11.
10. Blüher S, Panagiotou G, Petroff D, Markert J, Wagner A, Klemm T, et al. Effects of a 1-year exercise and lifestyle intervention on irisin, adipokines, and inflammatory markers in obese children. Obesity (Silver Spring). 2014;22(7):1701-8.
11. Jansen E, Mulkens S, Jansen A. Tackling childhood overweight: treating parents exclusively is effective. Int J Obes (Lond). 2011;35(4):501-9.
12. Skjåkødegård HF, Danielsen YS, Morken M, Linde SF, Kolko RP, Balantekin KN, et al. Study Protocol: A randomized controlled trial evaluating the effect of family-based behavioral treatment of childhood and adolescent obesity-The FABO-study. BMC Public Health. 2016;16(1):1106.
13. Ramalho S, Saint-Maurice PF, Silva D, Mansilha HF, Silva C, Gonçalves S, et al. APOLO-Teens, a web-based intervention for treatment-seeking adolescents with overweight or obesity: study protocol and baseline characterization of a Portuguese sample. Eat Weight Disord. 2020;25(2):453-63.
14. Croker H, Viner RM, Nicholls D, Haroun D, Chadwick P, Edwards C, et al. Family-based behavioural treatment of childhood obesity in a UK National Health Service setting: randomized controlled trial. Int J Obes (Lond). 2012;36(1):16-26.
15. Boutelle KN, Zucker NL, Peterson CB, Rydell SA, Cafri G, Harnack L. Two novel treatments to reduce overeating in overweight children: a randomized controlled trial. J Consult Clin Psychol. 2011;79(6):759-71.
16. Darling KE, Rancourt D, Evans EW, Ranzenhofer LM, Jelalian E. Adolescent Weight Management Intervention in a Nonclinical Setting: Changes in Eating-Related Cognitions and Depressive Symptoms. J Dev Behav Pediatr. 2021;42(7):579-87.
17. DeBar LL, Stevens VJ, Perrin N, Wu P, Pearson J, Yarborough BJ, et al. A primary care-based, multicomponent lifestyle intervention for overweight adolescent females. Pediatrics. 2012;129(3):e611-20.
18. Douglas SM, Hawkins GM, Berlin KS, Crouter SE, Epstein LH, Thomas JG, et al. Rationale and protocol for translating basic habituation research into family-based childhood obesity treatment: Families becoming healthy together study. Contemp Clin Trials. 2020;98:106153.
19. Doyle AC, Goldschmidt A, Huang C, Winzelberg AJ, Taylor CB, Wilfley DE. Reduction of overweight and eating disorder symptoms via the Internet in adolescents: a randomized controlled trial. J Adolesc Health. 2008;43(2):172-9.

20. Eichen DM, Strong DR, Rhee KE, Rock CL, Crow SJ, Epstein LH, et al. Change in eating disorder symptoms following pediatric obesity treatment. Int J Eat Disord. 2019;52(3):299-303.
21. Epstein LH, Paluch RA, Saelens BE, Ernst MM, Wilfley DE. Changes in eating disorder symptoms with pediatric obesity treatment. The Journal of Pediatrics. 2001;139(1):58-65.
22. Estabrooks PA, Shoup JA, Gattshall M, Dandamudi P, Shetterly S, Xu S. Automated telephone counseling for parents of overweight children: a randomized controlled trial. Am J Prev Med. 2009;36(1):35-42.
23. Follansbee-Junger K, Janicke DM, Sallinen BJ. The influence of a behavioral weight management program on disordered eating attitudes and behaviors in children with overweight. J Am Diet Assoc. 2010;110(11):1653-9.
24. Goldschmidt AB, Best JR, Stein RI, Saelens BE, Epstein LH, Wilfley DE. Predictors of child weight loss and maintenance among family-based treatment completers. J Consult Clin Psychol. 2014;82(6):1140-50.
25. Jelalian E, Mehlenbeck R, Lloyd-Richardson EE, Birmaher V, Wing RR. 'Adventure therapy' combined with cognitive-behavioral treatment for overweight adolescents. Int J Obes (Lond). 2006;30(1):31-9.
26. Raynor HA, Mazzeo SE, LaRose JG, Adams EL, Thornton LM, Caccavale LJ, et al. Effect of a High-Intensity Dietary Intervention on Changes in Dietary Intake and Eating Pathology during a Multicomponent Adolescent Obesity Intervention. Nutrients. 2021;13(6).
27. Saelens BE, Sallis JF, Wilfley DE, Patrick K, Cella JA, Buchta R. Behavioral weight control for overweight adolescents initiated in primary care. Obes Res. 2002;10(1):22-32.
28. Shomaker LB, Tanofsky-Kraff M, Matherne CE, Mehari RD, Olsen CH, Marwitz SE, et al. A randomized, comparative pilot trial of family-based interpersonal psychotherapy for reducing psychosocial symptoms, disordered-eating, and excess weight gain in at-risk preadolescents with loss-of-control-eating. Int J Eat Disord. 2017;50(9):1084-94.
29. Vidmar AP, Goran MI, Naguib M, Fink C, Wee CP, Hegedus E, et al. Time limited eating in adolescents with obesity (time LEAd): Study protocol. Contemp Clin Trials. 2020;95:106082.
30. Cheng HL, Griffin H, Claes B-E, Petocz P, Steinbeck K, Rooney K, et al. Influence of dietary macronutrient composition on eating behaviour and self-perception in young women undergoing weight management. Eating and Weight Disorders - Studies on Anorexia, Bulimia and Obesity. 2014;19(2):241-7.
31. Raman J, Hay P, Tchanturia K, Smith E. A randomised controlled trial of manualized cognitive remediation therapy in adult obesity. Appetite. 2018;123:269-79.
32. Rieger E, Treasure J, Murray K, Caterson I. The use of support people to improve the weight-related and psychological outcomes of adults with obesity: A randomised controlled trial. Behaviour Research and Therapy. 2017;94:48-59.
33. Seimon RV, Wild-Taylor AL, Keating SE, McClintock S, Harper C, Gibson AA, et al. Effect of weight loss via severe vs moderate energy restriction on lean mass and body composition among postmenopausal women with obesity: The tempo diet randomized clinical trial. JAMA Network Open. 2019;2(10).
34. Smith E, Whittingham C. Cognitive remediation therapy plus behavioural weight loss compared to behavioural weight loss alone for obesity: study protocol for a randomised controlled trial. Trials. 2017;18(1):42.
35. Zwickert K, Rieger E, Swinbourne J, Manns C, McAulay C, Gibson AA, et al. High or low intensity text-messaging combined with group treatment equally promote weight loss maintenance in obese adults. Obesity Research & Clinical Practice. 2016;10(6):680-91.
36. Bolognese MA, Franco CB, Ferrari A, Bennemann RM, Lopes SMA, Bertolini S, et al. Group Nutrition Counseling or Individualized Prescription for Women With Obesity? A Clinical Trial. Front Public Health. 2020;8:127.
37. Salvo V, Kristeller J, Montero Marin J, Sanudo A, Lourenço BH, Schveitzer MC, et al. Mindfulness as a complementary intervention in the treatment of overweight and obesity in primary health care: study protocol for a randomised controlled trial. Trials. 2018;19(1):277.
38. Moss EL, Tobin LN, Campbell TS, von Ranson KM. Behavioral weight-loss treatment plus motivational interviewing versus attention control: lessons learned from a randomized controlled trial. Trials. 2017;18(1):351.

39. Tanco S, Linden W, Earle T. Well-being and morbid obesity in women: A controlled therapy evaluation. International Journal of Eating Disorders. 1998;23(3):325-39.
40. Fogelholm M, Kukkonen-Harjula K, Oja P. Eating control and physical activity as determinants of short-term weight maintenance after a very-low-calorie diet among obese women. International Journal of Obesity. 1999;23(2):203-10.
41. Keränen A-M, Savolainen MJ, Reponen AH, Kujari M-L, Lindeman SM, Bloigu RS, et al. The effect of eating behavior on weight loss and maintenance during a lifestyle intervention. Preventive Medicine. 2009;49(1):32-8.
42. Hilbert A. Social facilitation maintenance treatment for adults with obesity: study protocol for a randomised-controlled feasibility study (SFM study). BMJ Open. 2016;6(8):e010845.
43. Christaki E, Kokkinos A, Costarelli V, Alexopoulos EC, Chrousos GP, Darviri C. Stress management can facilitate weight loss in Greek overweight and obese women: a pilot study. Journal of Human Nutrition and Dietetics. 2013;26(s1):132-9.
44. Dalle Grave R, Calugi S, Gavasso I, El Ghoch M, Marchesini G. A randomized trial of energy-restricted high-protein versus high-carbohydrate, low-fat diet in morbid obesity. Obesity. 2013;21(9):1774-81.
45. Muggia C, Falchi AG, Michelini I, Montagna E, De Silvestri A, Grecchi I, et al. Brief group cognitive behavioral treatment in addition to prescriptive diet versus standard care in obese and overweight patients. A randomized controlled trial. e-SPEN Journal. 2014;9(1):e26-e33.
46. Jospe MR, Roy M, Brown RC, Williams SM, Osborne HR, Meredith-Jones KA, et al. The Effect of Different Types of Monitoring Strategies on Weight Loss: A Randomized Controlled Trial. Obesity (Silver Spring). 2017;25(9):1490-8.
47. Boh B, Lemmens LHJM, Jansen A, Nederkoorn C, Kerkhofs V, Spanakis G, et al. An Ecological Momentary Intervention for weight loss and healthy eating via smartphone and Internet: study protocol for a randomised controlled trial. Trials. 2016;17(1):154.
48. Dassen FCM, Houben K, Van Breukelen GJP, Jansen A. Gamified working memory training in overweight individuals reduces food intake but not body weight. Appetite. 2018;124:89-98.
49. Schyns G, van den Akker K, Roefs A, Houben K, Jansen A. Exposure therapy vs lifestyle intervention to reduce food cue reactivity and binge eating in obesity: A pilot study. Journal of Behavior Therapy and Experimental Psychiatry. 2020;67:101453.
50. Werrij MQ, Jansen A, Mulkens S, Elgersma HJ, Ament AJ, Hospers HJ. Adding cognitive therapy to dietetic treatment is associated with less relapse in obesity. J Psychosom Res. 2009;67(4):315-24.
51. Podina IR, Fodor LA, Cosmoiu A, Boian R. An evidence-based gamified mHealth intervention for overweight young adults with maladaptive eating habits: study protocol for a randomized controlled trial. Trials. 2017;18(1):592.
52. Beaulieu K, Casanova N, Oustric P, Turicchi J, Gibbons C, Hopkins M, et al. Matched Weight Loss Through Intermittent or Continuous Energy Restriction Does Not Lead To Compensatory Increases in Appetite and Eating Behavior in a Randomized Controlled Trial in Women with Overweight and Obesity. The Journal of Nutrition. 2019;150(3):623-33.
53. Cooper Z, Doll HA, Hawker DM, Byrne S, Bonner G, Eeley E, et al. Testing a new cognitive behavioural treatment for obesity: A randomized controlled trial with three-year follow-up. Behaviour Research and Therapy. 2010;48(8):706-13.
54. Scott SE, Duarte C, Encantado J, Evans EH, Harjumaa M, Heitmann BL, et al. The NoHoW protocol: a multicentre 2×2 factorial randomised controlled trial investigating an evidence-based digital toolkit for weight loss maintenance in European adults. BMJ Open. 2019;9(9):e029425.
55. Simpson SA, McNamara R, Shaw C, Kelson M, Moriarty Y, Randell E, et al. A feasibility randomised controlled trial of a motivational interviewing-based intervention for weight loss maintenance in adults. Health Technol Assess. 2015;19(50):v-vi, xix-xxv, 1-378.
56. Whitelock V, Kersbergen I, Higgs S, Aveyard P, Halford JCG, Robinson E. A smartphone based attentive eating intervention for energy intake and weight loss: results from a randomised controlled trial. BMC Public Health. 2019;19(1):611.
57. Afari N, Herbert MS, Godfrey KM, Cuneo JG, Salamat JS, Mostoufi S, et al. Acceptance and commitment therapy as an adjunct to the MOVE! programme: a randomized controlled trial. Obes Sci Pract. 2019;5(5):397-407.

58. Ariel AH, Perri MG. Effect of dose of behavioral treatment for obesity on binge eating severity. Eating Behaviors. 2016;22:55-61.
59. Bacon L, Keim NL, Van Loan MD, Derricote M, Gale B, Kazaks A, et al. Evaluating a 'non-diet' wellness intervention for improvement of metabolic fitness, psychological well-being and eating and activity behaviors. Int J Obes Relat Metab Disord. 2002;26(6):854-65.
60. Barnes RD, Ivezaj V, Martino S, Pittman BP, Grilo CM. Back to Basics? No Weight Loss from Motivational Interviewing Compared to Nutrition Psychoeducation at One-Year Follow-Up. Obesity (Silver Spring). 2017;25(12):2074-8.
61. Boutelle KN, Eichen DM, Peterson CB, Strong DR, Rock CL, Marcus BH. Design of the PACIFIC study: A randomized controlled trial evaluating a novel treatment for adults with overweight and obesity. Contemp Clin Trials. 2019;84:105824.
62. Carels RA, Burmeister JM, Koball AM, Oehlhof MW, Hinman N, LeRoy M, et al. A randomized trial comparing two approaches to weight loss: differences in weight loss maintenance. J Health Psychol. 2014;19(2):296-311.
63. Carels RA, Caroline Miller J, Selensky JC, Hlavka R, Solar C, Rossi J, et al. Using an acceptance-based behavioral approach as a supplement to obesity treatment: A stepped-care approach. Journal of Contextual Behavioral Science. 2019;12:98-105.
64. Carpenter KM, Vickerman KA, Salmon EE, Javitz HS, Epel ES, Lovejoy JC. A Randomized Pilot Study of a Phone-Based Mindfulness and Weight Loss Program. Behavioral Medicine. 2019;45(4):271-81.
65. Dennis KE, Pane KW, Adams BK, Qi BB. The Impact of a Shipboard Weight Control Program. Obesity Research. 1999;7(1):60-7.
66. Dennis KE, Tomoyasu N, McCrone SH, Goldberg AP, Bunyard L, Bing Qi B. Self-Efficacy Targeted Treatments for Weight Loss in Postmenopausal Women. Sch Inq Nurs Pract. 2001(3):259-76.
67. DiMarco ID, Klein DA, Clark VL, Wilson GT. The use of motivational interviewing techniques to enhance the efficacy of guided self-help behavioral weight loss treatment. Eat Behav. 2009;10(2):134-6.
68. Glynn EL, Fleming SA, Edwards CG, Wilson MJ, Evans M, Leidy HJ. Consuming a Protein and Fiber-Based Supplement Preload Promotes Weight Loss and Alters Metabolic Markers in Overweight Adults in a 12-Week, Randomized, Double-Blind, Placebo-Controlled Trial. J Nutr. 2022;152(6):1415-25.
69. Goodrick GK, Poston WS, 2nd, Kimball KT, Reeves RS, Foreyt JP. Nondieting versus dieting treatment for overweight binge-eating women. J Consult Clin Psychol. 1998;66(2):363-8.
70. Jeffery RW, Hellerstedt WL, French SA, Baxter JE. A randomized trial of counseling for fat restriction versus calorie restriction in the treatment of obesity. Int J Obes Relat Metab Disord. 1995;19(2):132-7.
71. Kalarchian MA, Marcus MD, Courcoulas AP, Cheng Y, Levine MD. Preoperative lifestyle intervention in bariatric surgery: initial results from a randomized, controlled trial. Obesity (Silver Spring). 2013;21(2):254-60.
72. LaRose JG, Fava JL, Steeves EA, Hecht J, Wing RR, Raynor HA. Daily self-weighing within a lifestyle intervention: impact on disordered eating symptoms. Health Psychol. 2014;33(3):297-300.
73. Lillis J, Niemeier HM, Ross KM, Thomas JG, Leahey T, Unick J, et al. Weight loss intervention for individuals with high internal disinhibition: design of the Acceptance Based Behavioral Intervention (ABBI) randomized controlled trial. BMC Psychology. 2015;3(1):17.
74. Martin CK, Johnson WD, Myers CA, Apolzan JW, Earnest CP, Thomas DM, et al. Effect of different doses of supervised exercise on food intake, metabolism, and non-exercise physical activity: The E-MECHANIC randomized controlled trial. Am J Clin Nutr. 2019;110(3):583-92.
75. Mason C, de Dieu Tapsoba J, Duggan C, Wang CY, Alfano CM, McTiernan A. Eating behaviors and weight loss outcomes in a 12-month randomized trial of diet and/or exercise intervention in postmenopausal women. Int J Behav Nutr Phys Act. 2019;16(1):113.
76. Mensinger JL, Calogero RM, Tylka TL. Internalized weight stigma moderates eating behavior outcomes in women with high BMI participating in a healthy living program. Appetite. 2016;102:32-43.
77. Napolitano MA, Whiteley JA, Mavredes MN, Faro J, DiPietro L, Hayman LL, et al. Using social media to deliver weight loss programming to young adults: Design and rationale for the Healthy Body Healthy U (HBHU) trial. Contemp Clin Trials. 2017;60:1-13.
78. Pacanowski CR, Senso MM, Oriogun K, Crain AL, Sherwood NE. Binge eating behavior and weight loss maintenance over a 2-year period. J Obes. 2014;2014:249315.

79. Radin RM, Epel ES, Daubenmier J, Moran P, Schleicher S, Kristeller J, et al. Do stress eating or compulsive eating influence metabolic health in a mindfulness-based weight loss intervention? Health Psychol. 2020;39(2):147-58.
80. Ramirez EM, Rosen JC. A comparison of weight control and weight control plus body image therapy for obese men and women. J Consult Clin Psychol. 2001;69(3):440-6.
81. Raynor HA, Niemeier HM, Wing RR. Effect of limiting snack food variety on long-term sensory-specific satiety and monotony during obesity treatment. Eating Behaviors. 2006;7(1):1-14.
82. Smith BW, Shelley BM, Sloan AL, Colleran KM, Erickson K. A Preliminary Randomized Controlled Trial of a Mindful Eating Intervention for Post-menopausal Obese Women. Mindfulness. 2018;9:836-49.
83. Steinberg DM, Tate DF, Bennett GG, Ennett S, Samuel-Hodge C, Ward DS. Daily self-weighing and adverse psychological outcomes: a randomized controlled trial. Am J Prev Med. 2014;46(1):24-9.
84. Vander Wal JS, Waller SM, Klurfeld DM, McBurney MI, Cho S, Kapila M, et al. Effect of a post-dinner snack and partial meal replacement program on weight loss. Int J Food Sci Nutr. 2006;57(1-2):97-106.
85. Wadden TA, Foster GD, Letizia KA. One-year behavioral treatment of obesity: comparison of moderate and severe caloric restriction and the effects of weight maintenance therapy. J Consult Clin Psychol. 1994;62(1):165-71.
86. Wadden TA, Foster GD, Sarwer DB, Anderson DA, Gladis M, Sanderson RS, et al. Dieting and the development of eating disorders in obese women: results of a randomized controlled trial. Am J Clin Nutr. 2004;80(3):560-8.
87. Williamson DA, Martin CK, Anton SD, York-Crowe E, Han H, Redman L, et al. Is caloric restriction associated with development of eating-disorder symptoms? Results from the CALERIE trial. Health Psychol. 2008;27(1s):S32-42.
