# Supplementary material for: Eating disorders in weight-related therapy (EDIT): Protocol for a systematic review with individual participant data meta-analysis of eating disorder risk in behavioural weight management
Source: PLoS One. 2023 Jul 10;18(7):e0282401. doi: 10.1371/journal.pone.0282401 (PMC10332604; doi:10.1371/journal.pone.0282401)
Supplement: S3 Table — (PDF) [file pone.0282401.s003.pdf]

Table S3: Funding sources for trials which have agreed to join the EDIT Collaboration

Trial Author, year of publication	Funding
**ADOLESCENT TRIALS**	
Bonham et al. 2017	Jenny Craig Weight Loss Centers Pty Ltd (to M.B. and H.T.). The funders had no role in the design and conduct of the study; collection, management, analysis, and interpretation of the data; and preparation, review, or approval of the manuscript; nor the decision to submit the manuscript for publication. However, employees of Jenny Craig (consultants) were involved with the initial recruitment of participants and collection of anthropometric data.
Boutelle et al. 2011	University of Minnesota Faculty Development Grant to Kerri N. Boutelle and Lisa Harnack.
Braet et al. 2004	N/A
Cardel, Newsome et al. 2022	National Institute of Health National Heart, Lung, and Blood Institute K01HL141535.
Croker et al. 2012	Cancer Research UK, Great Ormond Street Hospital and Weight Concern.
Douglas et al. 2020	National Institutes of Health under award 1R01DK121360–01.
Eichen et al. 2019	National Institute of Diabetes and Digestive and Kidney Diseases, Grant/Award R01DK075861.
Epstein et al. 2001	N/A
Goldschmidt et al. 2014	NIH grants R01 HD036904, K24 MH070446, and T32 HL007456, and NCRR grants KL2 RR025000, KL2 RR024994, and UL1 RR024992. This research was supported in part by a Student Research Award by the American Psychological Association's Division 38 (Health Psychology) to Angela Celio Doyle, Ph.D., grant 1K24MH070446-02 from the National Institute of Mental Health, National Institutes of Health, awarded to Denise E. Wilfley, Ph.D., and an RGA/Washington University Longer Life Foundation Research Award to Dr. Wilfley.
Lister et al. 2020	Australian National Health and Medical Research Council (NHMRC) project grant funding 2017–2020 (#1128317).
Lofrano-Prado & Prado et al. 2017	National Council for Scientific and Technological Development - CNPQ and Foundation for Science and Technology of the State of Pernambuco – FACEPE.
Lofrano-Prado et al. 2021	Coordination for the Improvement of Higher Education Personnel (CAPES).
Partridge et al. 2020	NSW Health Early-Mid Career Researcher Grant under the NSW Cardiovascular Research Capacity Program.
Ramalho et al. 2020	Research was partially conducted at the Psychology Research Centre (PSI/01662), University of Minho, and supported by the Portuguese Foundation for Science and Technology and the Portuguese Ministry of Science, Technology and Higher Education through national funds, and co-financed by FEDER through COMPETE2020 under the PT2020 Partnership Agreement (POCI-01-0145-FEDER-007653), by the following grants to Eva Conceição (IF/01219/2014 and POCI-01-0145-FEDER-028209), and doctoral scholarship to Sofia Ramalho (SFRH/BD/104182/2014). The work from Pedro Saint-Maurice was partially funded by an individual fellowship grant awarded by the Fundação para a Ciência e a Tecnologia (FCT; Portugal) (SFRH/BI/114330/2016) under the POPH/FSE program.
Raynor et al. 2021	National Institute of Child Health and Human Development (R21HD084930) awarded to MKB. Additional support was obtained from the Children’s Hospital Foundation (unnumbered, to MKB and EPW) and the National Center for Advancing Translational Science (CTSA award UL1TR002649 to FGM). These funding agencies had no influence over the design or conduct of this work.

Shomaker et al. 2017	National Institutes of Health Intramural Research Program Grant 1ZIAHD000641 from NICHD (JAY). Pilot Intramural Research Award 72ON-01 from USUHS (MTK).
Skjåkødegård et al. 2016	Health Authorities of Western Norway and the University of Bergen. Accelerometers used in the trial were funded by a grant from the non-commercial organization Sov.no (Norwegian Competency Service for Sleep Disturbances). No commercial companies were funding any salaries or equipment for this trial.
Vidmar et al. 2020	This work was supported in part by grants (1) UL1TR001855 from the National Center for Advancing Translational Science (NCATS) of the U.S. National Institutes of Health, (2) NIH/NCRR SC-CTSI Grant Number UL1 TR000130, (3) National Institute on Minority Health and Health Disparities (NIMHD) Obesity Health Disparities Research Center (U54MD000502; Salvy/Dutton), the Eunice Kennedy Shriver National Institute of Child Health and Human Development (NICHD; R01HD092483; de la Haye/Salvy), and (4) the National Cancer Institute (NCI; 1R01CA258222, Figueiredo/Salvy/Peterson). The content is solely the responsibility of the authors and does not necessarily represent the official views of the National Institutes of Health. Dexcom supported this study by providing the continuous glucose monitor equipment required.
**ADULT TRIALS**	
Barnes et al. 2017	NIH career development awards, K23-DK092279 for RDB and K24-DK070052 for CMG.
Beaulieu et al. 2020	Funded by a Research Fellowship awarded to Kristine Beaulieu by the European Society for Clinical Nutrition and Metabolism (ESPEN) and in-kind support from LighterLife UK Ltd. The funding sources had no involvement in the conduct of the research and preparation of the article.
Bolognese et al. 2020	N/A
Boutelle et al. 2019	National Institutes of Health [R01DK103554, K23DK114480, UL1TR001442]. The content is solely the responsibility of the authors and does not necessarily represent the official views of the National Institutes of Health.
Carpenter et al. 2019	NCCIH/NIH grant 1R21AT007845-01A1. K Carpenter PI.
Cheng et al. 2014	Meat and Livestock Australia (Project No. D.MHN.0009) awarded to Dr Helen O'Connor. Meat and Livestock Australia had no role in the study design, collection, analysis, interpretation of data or writing of the paper including the decision to submit for publication
Dalle Grave et al. 2013	N/A
Dassen et al. 2018	Maastricht University Interfaculty Program 'Eatwell'
Hilbert et al. 2016	Grant 01EO1001 from the German Federal Ministry of Education and Research.
Jospe et al. 2017	SWIFT was funded the University of Otago (no grant number)
LaRose et al. 2014	R01DK074721 from the National Institute of Diabetes and Digestive and Kidney Diseases to HAR and K23DK083440 from the National Institute of Diabetes and Digestive and Kidney Diseases to JGL.
Martin et al. 2019	Supported by the NIH via the National Heart, Lung, and Blood Institute, with the Multiple Principal Investigators being CKM and TSC (R01 HL102166); NORC Center grant P30 DK072476, titled "Nutritional Programming: Environmental and Molecular Interactions," sponsored by the National Institute of Diabetes and Digestive and Kidney Diseases; the National Institute of General Medical Sciences, which funds the Louisiana Clinical and Translational Science Center (U54 GM104940); and NIH grant F32 HL123242. Grant R01 HL102166 was the main funding source for the project.
Mason et al. 2019	Funded in part through the NIH/NCI Cancer Center Support Grant P30 CA015704, and supported by grants from the National Cancer Institute at the National Institutes of Health: R01 CA105204-01A1, NIHR03 CA162482–01 and grants from the Breast Cancer Research Foundation (BCRF-16-106 and BCRF-17-105). The funding bodies had no role in the

	design of the study nor in the collection, analysis and interpretation of data, or manuscript preparation.
Mensinger et al. 2016	Grant awarded to the first author from the Edna G. Kynett Memorial Foundation. The Reading Hospital and Medical Center sponsored the project by holding the research at their site and paying the salary and benefits of the PI and Research Coordinators that went above and beyond the 85k in funding from the Kynett Foundation.
Pacanowski et al. 2014	Grant from the NIH/National Cancer Institute (R01 CA128211) and CP was supported by a University of Minnesota training grant (NIH T32 DK083250).
Raman et al. 2018	Diabetes Australia Research Trust Grant 2014.
Reiger et al. 2017	National Health and Medical Research Council of Australia project grant (#632621).
Salvo et al. 2018	Biochemistry examinations will be conducted by the São Paulo Municipal Health Department (in BHUs) and the remainder will be performed using own resources. The Brazilian Centre of Mindfulness and Health Promotion (Mente Aberta Centre) for subsidising the materials needed for this research (photocopy, electrodes for bioimpedance).
Seimon et al. 2019	Project Grant (1026005) from the National Health and Medical Research Council of Australia, awarded to A. Sainsbury, N. Byrne and I. Caterson. The Rebecca L. Cooper Medical Research Foundation and the University of Sydney/National Health and Medical Research Council of Australia provided grants that contributed to the purchase of equipment used for this trial. Prima Health Solutions (Brookvale, New South Wales, Australia) provided inkind support for this trial in the form of below-cost KicStart meal replacement products (shakes) and a gift of associated adherence tools (shakers). This relationship with Prima Health Solutions was established after the dietary protocol for the TEMPO Diet Trial had been established.
Simpson et al. 2015	National Institute for Health Research Health Technology Assessment programme.
Smith et al. 2017	Ramaciotti Foundation Australia (HIG2015/075) and Western Sydney University (Project code: 20311.64321).
Smith et al. 2018	Financial support from the La Tierra Sagrada Society of the University of New Mexico (UNM) and in-kind support from the UNM General Clinical Research Center.
Varady et al.	Trial is ongoing
Whitelock et al. 2019	Economic and Social Research Council [project reference: ES/N00034X/1]. The funding body had no role in the design of the trial, data collection, analysis and interpretation, or in writing the manuscript.
Williamson et al. 2008	National Institute on Aging, National Institutes of Health (U01AG022132, U01AG020478, U01AG020487, and U01AG020480).
Zwickert et al. 2016	Supported by the NHMRC via a Senior Research Fellowship to Amanda Sainsbury and by a Program Grant of the NHMRC APP1037786.
